# Unraveling Ros Conversion Through Enhanced Enzyme‐Like Activity with Copper‐Doped Cerium Oxide for Tumor Nanocatalytic Therapy

**DOI:** 10.1002/advs.202307154

**Published:** 2023-12-31

**Authors:** Zhengxiang Gu, Dan Zhong, Xingyu Hou, Xuelian Wei, Caikun Liu, Yechuan Zhang, Zhenyu Duan, Zhongwei Gu, Qiyong Gong, Kui Luo

**Affiliations:** ^1^ Department of Radiology Huaxi MR Research Center (HMRRC) Frontiers Science Center for Disease‐Related Molecular Network State Key Laboratory of Biotherapy West China Hospital Sichuan University Chengdu 610041 China; ^2^ National Engineering Research Center for Biomaterials Sichuan University 29 Wangjiang Road Chengdu 610064 China; ^3^ School of Chemistry and Materials Science Nanjing Normal University Nanjing 210023 China; ^4^ Functional and molecular imaging Key Laboratory of Sichuan Province and Research Unit of Psychoradiology Chinese Academy of Medical Sciences Chengdu 610041 China

**Keywords:** cerium oxide/copper, nanocatalytic therapy, nanozymes, oxygen vacancies, reactive oxygen species

## Abstract

Nanozyme catalytic therapy for cancer treatments has become one of the heated topics, and the therapeutic efficacy is highly correlated with their catalytic efficiency. In this work, three copper‐doped CeO_2_ supports with various structures as well as crystal facets are developed to realize dual enzyme‐mimic catalytic activities, that is superoxide dismutase (SOD) to reduce superoxide radicals to H_2_O_2_ and peroxidase (POD) to transform H_2_O_2_ to ∙OH. The wire‐shaped CeO_2_/Cu‐W has the richest surface oxygen vacancies, and a low level of oxygen vacancy (Vo) formation energy, which allows for the elimination of intracellular reactive oxygen spieces (ROS) and continuous transformation to ∙OH with cascade reaction. Moreover, the wire‐shaped CeO_2_/Cu‐W displays the highest toxic ∙OH production capacity in an acidic intracellular environment, inducing breast cancer cell death and pro‐apoptotic autophagy. Therefore, wire‐shaped CeO_2_/Cu nanoparticles as an artificial enzyme system can have great potential in the intervention of intracellular ROS in cancer cells, achieving efficacious nanocatalytic therapy.

## Graphical abstract

A high degree of surface oxygen mobility and a low level of oxygen vacancy formation energy is exposed over the CeO_2_/Cu‐W surface, which is employed as a mimetic enzyme of superoxide dismutase (SOD) to convert O_2_^∙‐^ →H_2_O_2_ and peroxidase (POD) to realize H_2_O_2_→∙OH for oncotherapy.

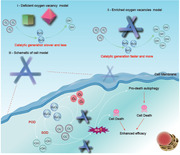

## 1 Introduction

Despite the development of multiple anti‐cancer strategies, chemotherapy is still an indispensable therapeutic method for different malignant tumors.^[^ ^1^, ^2^ ^]^ Nanozyme catalytic therapy is an emerging chemotherapeutic method. Nanozymes, nanomaterials with enzyme‐mimicking properties, have drawn increasing attention due to their advantages such as improved stability, higher cost‐effectiveness, and less strict storage conditions in contrast with natural enzymes.^[^ ^3^, ^4^ ^]^ Particularly, incorporating their distinctive properties and catalytic abilities, nanozymes have great potential in various applications, ranging from diagnosis in vitro to substitutive use in certain biosystems.^[^ ^5^, ^6^ ^]^ Nanozymes have been explored for cancer treatment by modulating intracellular reactive oxygen species (ROS).^[^ ^7^ ^]^

ROS is essential for living organisms due to its important function in regulating various physiological functions. The intrinsic biochemical properties of ROS can demonstrate mechanisms for the physiological activities of living organisms, which are worth being studied in depth.^[^ ^8^, ^9^ ^]^ In cancer cells, intracellular electron transport chains and NADPH oxidases result in continuous generation of O_2_^∙−^.^[^ ^10^ ^]^ O_2_^∙‐^ can be steadily converted into H_2_O_2_ catalyzed by superoxide dismutase (SOD) and be further transformed to hydroxyl radicals through the peroxidase‐like (POD) reaction.^[^ ^11^ ^]^ To improve the catalytic ability of nanozyme, Ir‐N_5_ SA/Cer was prepared by introducing axial N coordination in SA enzymes. It has been reported that the Ir‐N_5_ SA can activate natural oxidase, peroxidase, and catalase, as well as catalyzing NAHD to H_2_O_2._^[^ ^12^ ^]^ However, it is challenging to promote the production of ∙OH by intracellular SOD and POD, thus, constant production of ∙OH inside cancer cells has been attempted by various approaches to enhance the cytotoxicity of ROS for anti‐tumor treatment.^[^ ^13^ ^]^ Nanozymes could be developed to continuously convert endogenous ROS (O_2_^∙−^ and H_2_O_2_) into ∙OH inside cancer cells.

**Figure 1:**
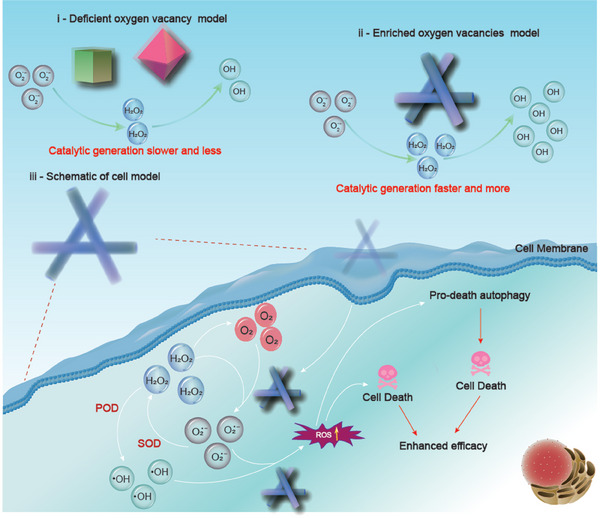
The mechanism for CeO_2_/Cu to mediate the relay transformation of O_2_^∙‐^→H_2_O_2_→OH to induce cancer cell death.

CeO_2_ possesses sufficient surface oxygen vacancies, which are critical for converting O_2_^∙−^ hydrogenation and H_2_O_2_ resolving.^[^ ^14^ ^]^ CeO_2_ with morphology of different exposed crystal facets has been found to be essential in a series of reactions. Herein, we have fabricated CeO_2_ of wires (CeO_2_/Cu‐W), cubes (CeO_2_/Cu‐C), and octahedrons (CeO_2_/Cu‐O) as the support for the Cu metal. The CeO_2_/Cu‐W exhibits a high SOD and POD activity in contrast with CeO_2_/Cu‐C and CeO_2_/Cu‐O and facilitates continuous catalysis of O_2_^∙−^ → H_2_O_2_ → ∙OH in cancer cells via the relay reaction supported by oxygen vacancies, eventually achieving constant production of ∙OH, triggering the apoptosis of cancer cells (**Figure** **1**).

## 2 Results and Discussion

**Figure 2:**
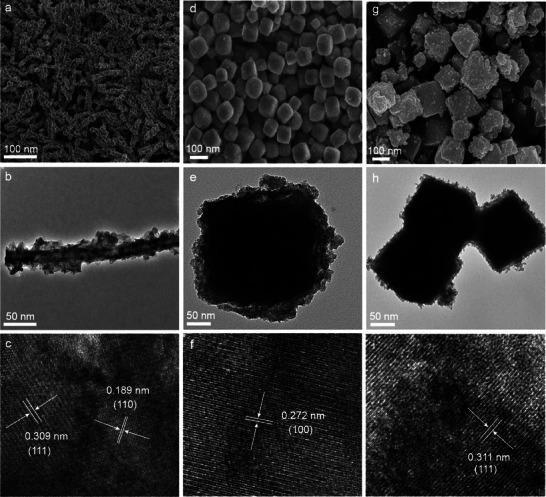
SEM and TEM images of CeO_2_/Cu‐W a–c), CeO_2_/Cu‐C d–f), and CeO_2_‐C g–i).

To verify the crystal characteristics of CeO_2_ and CeO_2_/Cu, powder X‐ray diffraction (XRD) experiments were conducted. Figure S1 (Supporting Information) displays the XRD patterns of CeO_2_ and CeO_2_/Cu with different morphologies. Scanning electron microscopy (SEM) images show that CeO_2_ with different shapes are uniformly arranged on the substrate with a smooth surface (Figure S2, Supporting Information), indicating the well‐prepared CeO_2_ works as a template for the following reaction. Transmission electron microscopy (TEM) images in Figure S3 (Supporting Information) exhibit the same smooth surface as SEM images. Mapping analysis by Energy‐dispersive X‐ray spectroscopy verifies uniform distribution of Cu, Ce, and O in CeO_2_/Cu, indicating that Cu components are doped into nanostructure rather than coating, exhibited in Figure S4 (Supporting Information). **Figure** **2** shows TEM and SEM images, which demonstrate the exposed lattice plane of CeO_2_/Cu samples with different morphologies. For wire‐shaped CeO_2_/Cu‐W (Figure 2a–c), which have a length of 100–300 nm and a width of ≈10 nm, and the interplanar spacing in CeO_2_/Cu‐W is 0.189 and 0.309 nm, corresponding to (110) and (111) crystal plane. CeO_2_/Cu‐C has a shape of cubes at a bulk with 200 nm and the (100) crystal facet exhibits a distinct interplanar spacing of 0.272 nm, revealed by Figure 2d,f. Based on Figure 2g, CeO_2_/Cu‐O particles exhibit a morphology of octahedrons with a length of 200–300 nm. The (111) facet with an interplanar spacing of 0.311 nm is selectively exposed due to its thermodynamical stability (Figure 2i).

**Figure 3:**
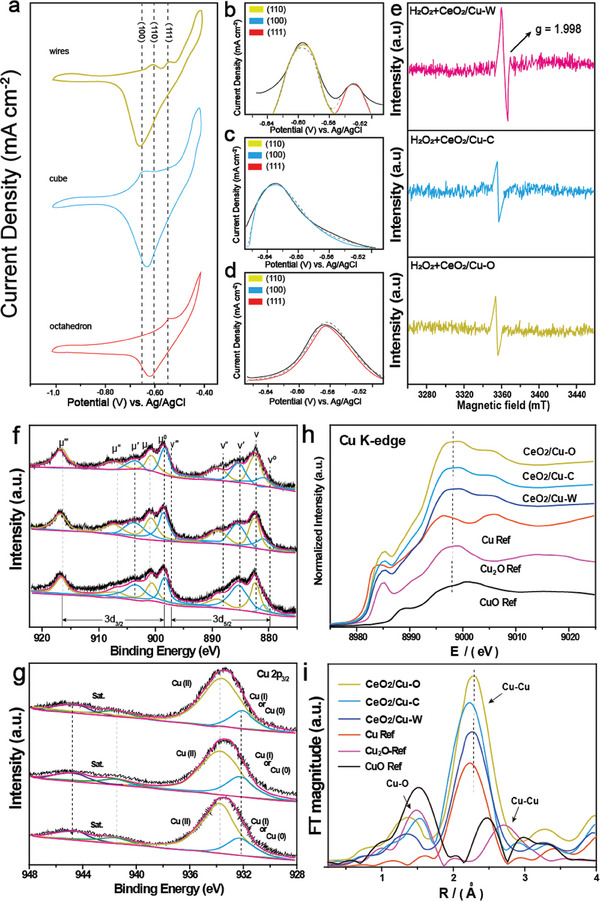
a) C–V curves of NWs, NPs, and NSs in Ar‐saturated KOH (1.0m) at a scanning rate of 50 mV s^−1^. b–d) The OH^−^ adsorption peaks of the CeO_2_/Cu‐W (b), CeO_2_/Cu‐C c), and CeO_2_/Cu‐O d) were fitted to the corresponding crystal plane proportions in the inset. e) EPR spectra of CeO_2_/Cu‐W, CeO_2_/Cu‐C and CeO_2_/Cu‐O. f,g) XPS characterization of Ce 3d f) and Cu 2p_3/2_ g) of CeO_2_/Cu. h) XANES spectra at the Cu K‐edge. i) The corresponding K^3^‐weighted Fourier‐Transform (FT) spectra in R‐space.

The selectivity by crystal facets was confirmed by the electro‐sorption of hydroxide (OH_ads_) studies.^[^ ^15^ ^]^ Cyclic voltammetry results, as shown in **Figure** **3**a, indicated that different facets exhibit different OH^−^ adsorption/desorption peaks in the C–V curves caused by the exposed crystal facets of CeO_2_/Cu catalysts with different morphologies.^[^ ^16^ ^]^ Peaks at −0.64, −0.59, and −0.54 V_Ag/AgCl_ in the C–V curves represent (100), (110), and (111) planes, respectively. In addition, three distinguished adsorption peaks can be found, which indicates a similar distribution trend on the surface of catalysts. The relative ratio of the crystal planes could be obtained by the integration of three adsorption peaks (Figure 3b–d). To further illustrate the existence of oxygen vacancies, electron paramagnetic resonance spectra were obtained (Figure 3e). As a result, CeO_2_/Cu‐C and CeO_2_/Cu‐O present weakly unpaired electrons, while CeO_2_/Cu‐W exhibits a stronger opposite peak at *g* = 1.998, and the increased intensity of the opposite peak is ascribed to the increase in the oxygen vacancy density, suggesting that CeO_2_/Cu‐W has the richest surface oxygen vacancies.^[^ ^17^ ^]^ The actual Cu/Ce ratio in different samples can be confirmed using an inductively coupled plasma mass spectrometry (ICP‐MS) (Table S1, Supporting Information).

The valence states of Ce and Cu was investigated using an X‐ray photoelectron spectroscopy (XPS). Figure 3f indicates that Ce 3d can be fitted into 10 peaks, which corresponds to the Ce 3d_5/2_ and Ce 3d_3/2_ states.^[^ ^18^ ^]^ The characteristic peaks of ν_o_, ν′, µ_o_, and µ‴ are generated due to the existence of Ce^3+^ species while other six peaks can be ascribed to Ce^4+^ species.^[^ ^19^ ^]^ The surface density of Ce^3+^ can be determined according to Ce^4+^ and Ce^3+^ peaks areas. The ratios of Ce^3+^ and Ce^4+^ species regarding all obtained samples are showed in Table S2 (Supporting Information). The Ce^3+^ concentration is related to the surface oxygen vacancies, and their formation is associated with the existence of Ce^3+^ species.^[^ ^20^ ^]^ Our calculations suggest that the correlation could be challenged by the Cu^2+^ substitution. In addition, the substitution of one Ce^3+^ next to a V_O_ by one Cu^2+^ can be due to the conversion of Ce^3+^ to Ce^4+^. Consequently, an increase in the Cu^2+^ doping content leads to a decline in the Ce^3+^/Ce^4+^ ratio of the initial CeO_2_, which is in accordance with lower copper proportion from XPS data. CeO_2_/Cu‐W exhibits the highest concentration of Ce^3+^ (0.49) among all samples while the CeO_2_/Cu‐C and CeO_2_/Cu‐O are 0.38 and 0.36, respectively. Therefore, the assumption that surface oxygen vacancies of CeO_2_/Cu‐W possess the highest density is made. The peaks of 933.6 and 932.5 eV in spectra of Cu 2p_3/2_ were assigned to Cu^2+^ and [Cu^0^+Cu^1+^] (Figure 3g).^[^ ^21^ ^]^ The low oxidation potential of [Cu^0^+Cu^1+^] suggest that oxygen vacancies are generated around the doping sites of Cu atoms.^[^ ^22^ ^]^ The concentration of the oxygen vacancy can be determined by O 1s spectra. As depicted in Figure S5 (Supporting Information), deconvoluted result showed three peaks at 529.3, 531.4, and 534.0 eV, of which 529.3 and 531.4 eV demonstrated the existence of lattice oxygen (O_α_: O^−^) and surface oxygen (O_β_: O^−^, O_2_^2−^ or O^2‐^), respectively. Meanwhile, the peak at 534.0 eV verifies other oxygen species (O_γ_) with weak bound including CO_3_^2−^, adsorbed H_2_O, and OH^−^.^[^ ^23^ ^]^ The abundance of oxygen vacancies (V_O_) can be calculated based on the ratio of O_β_ and (O_α_+O_β_+O_γ_), in line with Ce 3d spectra. The above results indicates that the amount of surface oxygen vacancies is proportional to the number of Ce^3+^. It could be suggested that CeO_2_ with different morphologies exposes different crystal facets that possess distinct V_O_ formation energy, thereby exhibiting various densities of V_O_.

The atomic structure of obtained samples was confirmed by X‐ray absorption near‐edge structure (XANES) and extended X‐ray absorption fine structure (EXAFS) (Figure 3h,i). In contrast with standard CuO sample (CuO‐ref, black curve), the peaks of all Cu‐doped CeO_2_ samples display a distinct shift toward the lower energy side, which results from the low oxidative potential of [Cu^0^+Cu^1+^]. The characteristic peak shapes of the three samples are similar to those of standard Cu_2_O (Cu_2_O‐ref, purple curve) at ≈899.6 eV. The XANES spectrum of CeO_2_/Cu is between two standard spectra, indicating the coexistence of +1 and +2 valence states of Cu atoms. The conversion of EXAFS at the Cu *K*‐edge to *R*‐space (Figure 3i) gives more structural information of the three samples. The three peaks in dotted lines represent the Cu─O and Cu─Cu bonds. Compared to standard Cu and Cu_2_O samples, the three samples exhibit a main peak at 2.14 Å (Cu─Cu bond) and a minor one at 1.55 Å, indicating a high percentage of Cu─Cu bonds and a low percentage of Cu─O bonds. In Figure S6 (Supporting Information), the coordination peak of the Cu─Cu bond of CeO_2_/Cu‐C and CeO_2_/Cu‐O centered at 2.12 and 2.15 Å. However, CeO_2_/Cu‐W exhibited a shift for that of the Cu─Cu bond at 2.14 Å, which could be attributed to the generation of oxygen vacancies. The enhanced oxygen vacancies of the three samples indicate that it can significantly enhance the chemocatalysis capability of O_2_^∙−^.^[^ ^24^ ^]^ Furthermore, the Cu LMM Auger spectrum was utilized to determine the oxidation state of copper for the CeO_2_/Cu‐W sample. Two final‐state terms split from the L−S coupling ^1^G and ^3^F have been previously reported. The ^1^G peaks are detected for different valence states of Cu: 917.1 (Cu^2+^), 915.8 (Cu^1+^), and 918.0 eV (Cu^0^), while the ^3^F peak represents the existence of Cu^0^.^[^ ^25^ ^]^ Displayed in Figure S7 (Supporting Information), the ^1^G peak at 916.5 eV of CeO_2_/Cu‐W suggests that Cu is in a valence state of between +1 and +2. On the other hand, the distinct ^3^F peak confirms the presence of Cu^0^, which corresponds to the XAS results.

**Figure 4:**
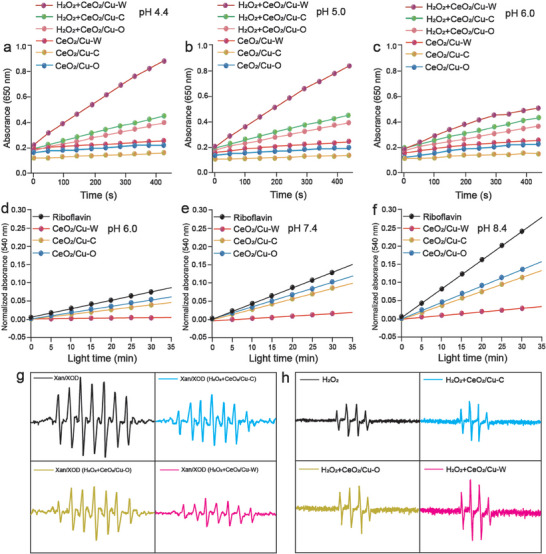
a–c) Temporal absorbance variations at a wavelength of 650 nm in different shaped CeO_2_/Cu/TMB/H_2_O_2_ systems at pH of 4.4, 5.0, and 6.0. d–f) Normalized temporal absorbance variations at 540 nm wavelength for different samples. g,h) ESR spectra in two different solution systems.

The catalytic capability of SOD and POD is essential in catalyzing the endogenous peroxides and superoxide into hydroxyl radicals. The SOD and the POD units processed by CeO_2_/Cu work in concert to achieve the cascade relay catalysis that promotes the serial transformation of O_2_^∙−^ → H_2_O_2_ → ∙OH.^[^ ^26^ ^]^ The POD‐like enzymatic capability of CeO_2_/Cu at pH 4.4, 5.0, and 6.0 were measured via the classic H_2_O_2_/TMB system. CeO_2_/Cu‐W possesses a higher and more sustainable POD‐like activity at different pH levels than CeO_2_/Cu‐C and CeO_2_/Cu‐O according to the time course curves, also evidenced by a higher concentration of ∙OH based on the absorbance value at 650 nm (**Figure** **4**a–c; Figure S8, Supporting Information).^[^ ^27^ ^]^ To examine the SOD‐like activity of CeO_2_/Cu samples, the experiment of O_2_^∙−^/NBT (Nitrobluetetrazolium) inhibition assay was conducted.^[^ ^28^ ^]^ The generation of O_2_^∙−^ is triggered by riboflavin illumination, which can be detected by the NBT, and the formation of NBT‐monoformazan can be revealed by the absorption peak at 540 nm. Due to the SOD‐like activity of CeO_2_/Cu‐W, the reduced level of O_2_^∙‐^ results in a reduction in the absorption value at 540 nm. However, the CeO_2_/Cu‐C and CeO_2_/Cu‐O samples have a negligible effect on the absorption value. At pH 6.0 and 7.4, riboflavin illumination results in a continuous increase in the absorbance with the addition of the illumination time (Figure S9, Supporting Information). Nevertheless, CeO_2_/Cu‐W curbs the increase of the absorbance to a great extent compared to CeO_2_/Cu‐C and CeO_2_/Cu‐O, suggesting its superior SOD‐like activity (Figure 4d–f). The above experimental results confirm the superior POD‐ and SOD‐like activities of CeO_2_/Cu‐W that can improve the catalytic efficiency of the reduction process. The xanthine/xanthine oxidase system (Xan+XOD) was also employed to verify the catalytic activity of CeO_2_/Cu‐W.^[^ ^29^ ^]^ Similar to the results of inhibition assay of O_2_^∙−^/NBT, the CeO_2_/Cu‐W/(Xan+XOD)/DMPO system exhibits a weaker ESR signal of DMPO/ O_2_^∙−^, indicating that CeO_2_/Cu‐W can catalyze O_2_^∙−^ to H_2_O_2_ more efficiently (Figure 4g). The stronger ESR signal of DMPO/∙OH using the CeO_2_/Cu‐W/H_2_O_2_/DMPO system suggests that CeO_2_/Cu‐W promotes the catalytic process of H_2_O_2_ → ∙OH at pH = 6.0 (Figure 4h). Therefore, it can be concluded that CeO_2_/Cu‐W can facilitate the overall reaction of O_2_^∙‐^ → H_2_O_2_→ ∙OH to achieve a better tumor inhibition efficacy.

**Figure 5:**
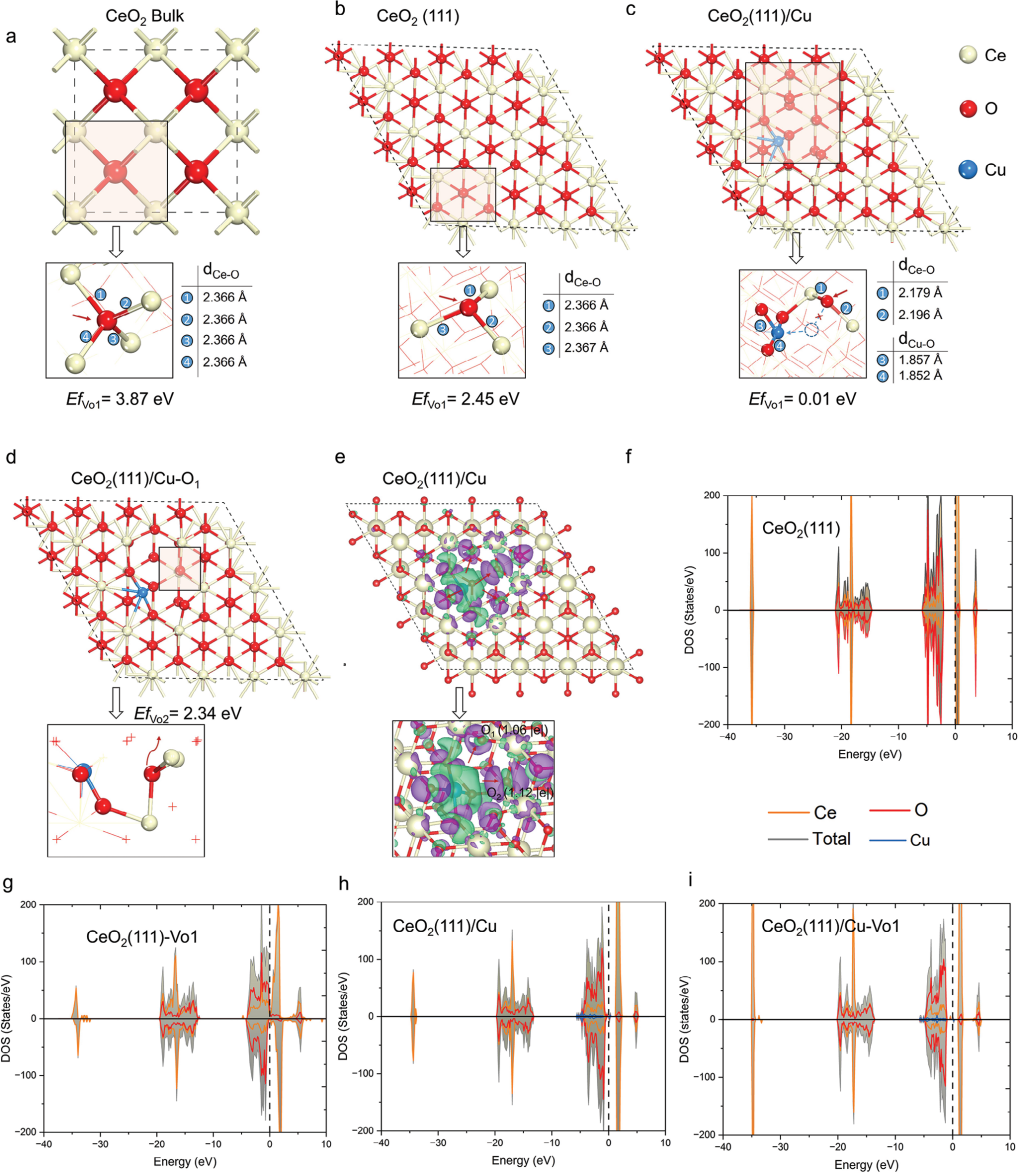
DFT calculations for analyzing the mechanism of O vacancies formation in the CeO_2_. a) Optimized geometry of the CeO_2_ bulk. Key Ce─O bonds and O vacancy formation energies are displayed. b) The geometry of CeO_2_(111) can be optimized by using the Ce─O bond length and the oxygen vacancy formation of surface oxygen atoms. c) Optimized geometry of CeO_2_(111)/Cu with Ce‐O, Cu─O bond lengths, and oxygen vacancy formation energies of the oxygen atom in the surface layer. d) Optimized geometry of CeO_2_(111)/Cu‐Vo1 with the oxygen vacancy formation energy of the oxygen atom in the subsurface layer. e) Charge density differences of CeO_2_(111)/Cu. The Bader charge of the O atoms in the surface and subsurface of CeO_2_(111) are shown. f–i). Density of states (DOS) of Ce(111), CeO_2_(111)‐Vo1, CeO_2_(111)/Cu, and CeO_2_(111)/Cu‐Vo1, respectively.

We further reveal the mechanism of oxygen vacancy formation through DFT calculations and elucidate that Cu doping can effectively promote the oxygen supply capacity of CeO_2_. Previous theoretical and experimental results suggested that CeO_2_ (111) has a lower surface energy and is more likely to form stable structures, which is consistent with our experimental results.^[^ ^30^ ^]^ The effect of the Cu‐doped CeO_2_(111) surface on the formation of oxygen vacancies was then examined. **Figure** **5**a plots the optimized CeO_2_ bulk at a relaxed Ce─O bond length of 2.366 Å. The O vacancy formation energy in the stable CeO_2_ bulk is 3. 87 eV. Although the coordination number of the O atom on the surface of CeO_2_(111) is decreased to three, the bond length of the Ce─O remains unchanged (Figure 5b). It can be attributed to the dangling bonds on the CeO_2_(111). However, the forming energy of oxygen vacancy on the surface of CeO_2_(111) is only 2.45 eV.

It is worth noting that the doped Cu atom can effectively lower the oxygen vacancy formation energy. Figure 5c shows the doped Cu atom is unsettled, leading to the breakage of the Cu─O bond. The passive oxygen atom on the surface of CeO_2_(111) has to shorten the residual Ce─O bond length (2.179, 2.196 Å) to maintain structural stability. The oxygen atoms on the surface have a lower coordination number (2), leading to an ultra‐low oxygen vacancy formation energy of 0.01 eV. Thus, the unknotted O atoms are more likely to migrate, providing rapid supply of oxygen to the surface of CeO_2_(111). Moreover, the defect structure caused by Cu doping enables the oxygen atoms in the subsurface layer to have a low oxygen defect formation energy. From Figure 5d, the oxygen vacancy formation energy is 2.34 eV, which is 0.11 eV smaller than that of the O atom on the surface of Ce (111). The charge density difference of CeO_2_(111)/Cu (Figure 5e) exhibits that the doped Cu atom transfers electrons to the oxygen atom, resulting in the reduction of the oxygen atoms. In addition, it can be seen from the density of states (DOSs) (Figure 5f–i) that the doping of the Cu atom leads to the gain of electrons by the antibonding orbitals of oxygen atoms to reach the Fermi energy level, which finally causes the activation of oxygen atoms.

**Figure 6:**
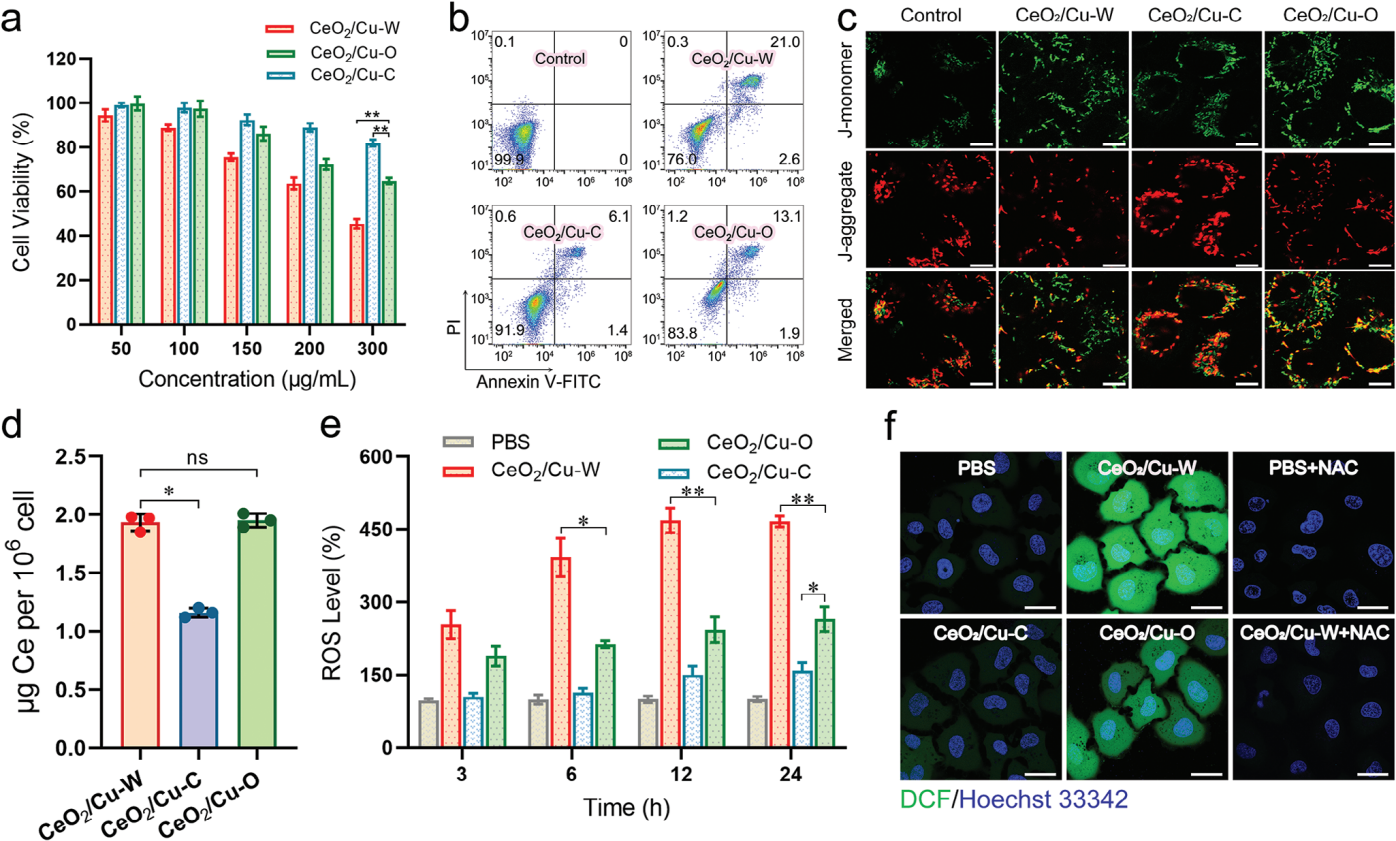
a) Cell viability of MDA‐MB‐231 cells after incubation with different CeO_2_/Cu nanoparticles for 24 h (*n* = 5, ^**^ *p* < 0.001). b) cell apoptosis and c) mitochondrial membrane potential changes of MDA‐MB‐231 cells after various treatments for 24 h. (Scale bar:10 µm). d) Intracellular Ce in MDA‐MB‐231 cells after co‐incubation with CeO_2_/Cu‐W, CeO_2_/Cu‐C or CeO_2_/Cu‐O (50 µg mL^−1^) for 6 h (*n* = 3, ^*^ *p* < 0.01, ns nonsignificant). e) Quantitative analysis of intracellular ROS after exposure to CeO_2_/Cu nanoparticles for different durations (*n* = 3). PBS‐treated cells served as a control. ^**^ *p* < 0.01^*^ *p* < 0.05. f) CLSM images of intracellular ROS in cells after incubation with CeO_2_/Cu nanoparticles for 12 h. DCF fluorescence (green) for intracellular ROS and Hoechst 33342 (blue) for cell nuclei. (Scale bar:20 µm). Data was expressed as mean ± SD. The *P* values were calculated using a one‐way ANOVA with Tukey's post‐hoc test a,d,e).

In view of the efficient catalytic properties of CeO_2_/Cu‐W nanoparticles O_2_^∙−^ → H_2_O_2_ → ∙OH, we studied whether they have a potential cytotoxic effect on tumor cells. The cytotoxicity of the CeO_2_/Cu nanoparticles was determined by CCK‐8 assays. As the concentration of CeO_2_/Cu nanoparticles increases, the viabilities of human breast carcinoma MDA‐MB‐231 cells showed a general decrease in a dose‐dependent manner (**Figure** **6**a). At a concentration of 300 µg mL^−1^, CeO_2_/Cu‐W treatment results in more than 50% decrease in the cell viability, which is much higher than CeO_2_/Cu‐C (18%) and CeO_2_/Cu‐O (35%). The similar killing effect of CeO_2_/Cu‐W nanoparticles is identified in 4T1 and A549 cell lines (Figures S10 and S11, Supporting Information). Consistent with the results of cell viabilities, the employment of CeO_2_/Cu‐W nanoparticles leads to the highest ratio of cell apoptosis (Figure 6b) and significant loss of the mitochondria membrane potential (MMP, Figure 6c).

In order to investigate whether the difference in the level of cell apoptosis induced by these nanoparticles is attributed to the number of internalized nanoparticles, the intracellular Ce concentration was determined by ICP‐MS. The intracellular content of Ce in CeO_2_/Cu‐W‐treated cells is similar to that in the CeO_2_/Cu‐O‐treated cells but slightly higher than that in the CeO_2_/Cu‐C‐treated cells (Figure 6d), indicating that the shape of CeO_2_/Cu‐W and CeO_2_/Cu‐O nanoparticles have limited effect on their cellular internalization, and the stronger antitumor effect of CeO_2_/Cu‐W than CeO_2_/Cu‐O is not due to the level of cellular internalization.

Then, the intracellular level of ROS (including O_2_^∙−^, H_2_O_2_ and ∙OH) was examined by a microplate reader using DCFH‐DA. After 12 h incubation, the relative ROS level in the CeO_2_/Cu‐W‐treated cells is three times of that in the CeO_2_/Cu‐C‐treated cells (^**^ *p*<0.01) and two times of that in the CeO_2_/Cu‐O‐treated cells (^**^ *p*<0.01), respectively (Figure 6e). Furthermore, the cells exposed to CeO_2_/Cu‐W display the strongest green fluorescence in the images captured by confocal laser scanning microscopy (CLSM) (Figure 6f), indicating that CeO_2_/Cu‐W has the strongest ROS production ability, in agreement with the results shown in Figures 4, 5. However, after the cells are pretreated with *N*‐acety‐L‐cysteine (NAC), a ROS scavenger, the intracellular green fluorescence intensity drastically decreases (Figure 6f; Figure S12, Supporting Information). ROS plays important roles in diverse biological processes, but a high level of ROS usually causes cell damage and induces autophagy.

**Figure 7:**
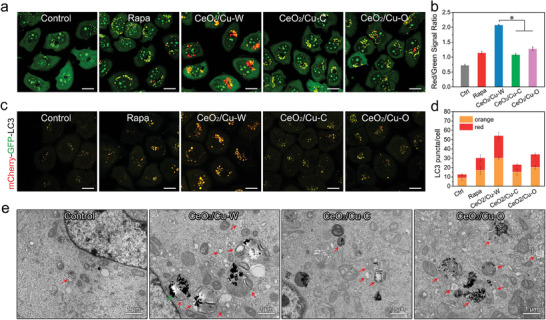
Analysis of intracellular autophagic levels activated by nanoparticles. a) CLSM images and b) FACS analysis of AO‐stained MDA‐MB‐231 cells after exposure to Rapa (50 nm), CeO_2_/Cu‐W, CeO_2_/Cu‐C or CeO_2_/Cu─O (100 µg mL^−1^) for 24 h (*n* = 3, ^*^ *p* < 0.01). c) Representative CLSM images of mCherry‐GFP‐LC3‐expression cells and d) Quantification of LC3 puncta per cell after incubation with different formulations for 24 h. e) TEM images for ultrastructural analysis of cells receiving treatment with PBS, CeO_2_/Cu‐W, CeO_2_/Cu‐C, or CeO_2_/Cu‐O (100 µg mL^−1^) for 24 h (red arrows: typical autophagic vacuoles; green arrow: CeO_2_/Cu nanoparticles).

Accordingly, the ability of CeO_2_/Cu‐W to induce autophagy in MDA‐MB‐231 cells was evaluated by acridine orange (AO), an autophagy probe, which appears red in acidic organelles (autophagosomes and lysosomes) but green in the cytosol and nuclei. The number of red spots counted in CLSM images has positive correlation with the concentration of nanoparticles (Figure S13, Supporting Information). The maximum number of red spots is found in the cells after treatment with CeO_2_/Cu‐W (100 µg mL^−1^) for 24 h, indicating that CeO_2_/Cu‐W has the strongest autophagy induction ability (**Figure** **7**a). Accordingly, the red to green signal ratio in the cells treated with CeO_2_/Cu‐W determined by fluorescence‐activated cell sorter (FACS) is the highest among all groups (Figure 7b), and it elevated significantly compared to that in the cells treated with CeO_2_/Cu‐C, CeO_2_/Cu‐O and rapamycin (Rapa, a clinical autophagy inducer), demonstrating the strong ability of CeO_2_/Cu‐W to induce production and accumulation of acidic organelles. But AO is able to differentiate whether the labeled organelles are autophagosomes and autolysosomes. Thus, mCherry‐GFP‐LC3‐expressed MDA‐MB‐231 cells were utilized to monitor the autophagic process induced by different shaped nanoparticles.

LC3, a specific autophagy‐related protein, aggregates on the autophagosome membranes when autophagy occurs. mCherry, an acid‐stable protein, shows red fluorescence in both autophagosomes and autolysosomes, while GFP is an acid‐unstable protein and its green fluorescence quenches after autophagosomes fuse with lysosomes to form autolysosomes. As shown in Figure 7c,d, CeO_2_/Cu‐W exposure leads to a significant increase in red and large yellow puncta in comparison with CeO_2_/Cu‐C and CeO_2_/Cu‐O, indicating that CeO_2_/Cu‐W could effectively stimulate the autophagic flux in cells. Finally, the bio‐TEM method was used to directly observe the autophagy level (Figure 7e). Compared to the PBS‐treated control cells, cells treated with CeO_2_/Cu‐W reveal abnormally accumulated autophagic vacuoles, while a small number of autophagic vacuoles is detected in the cells treated with CeO_2_/Cu‐C and CeO_2_/Cu‐O. These results are in consistence with the observations by confocal fluorescence imaging, providing support for the conclusion that the ability of eliciting autophagy by CeO_2_/Cu nanoparticles is shape‐dependent, and CeO_2_/Cu‐W elicits a high level of autophagy in the MDA‐MB‐231 cancer cells. As the upstream of autophagy. Indeed, the elimination of ROS by NAC could attenuate the CeO_2_/Cu‐W‐induced autophagy (Figure S14, Supporting Information).

**Figure 8:**
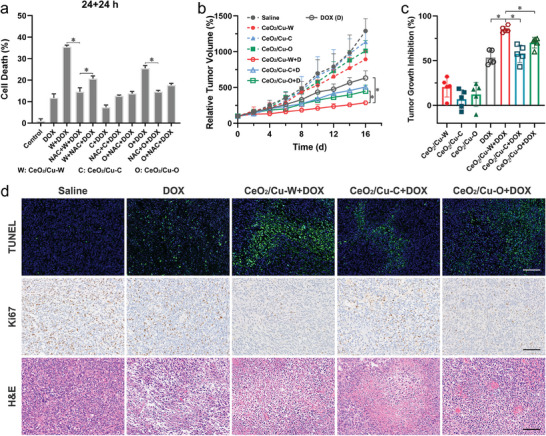
Antitumor efficiency of the nanoparticles. a) Cell mortality of MDA‐MB‐231 cells treated with different combinations for 48 h (*n* = 5, ^**^ *p* < 0.01, ^*^ *p* < 0.05). CeO_2_/Cu nanoparticles: 100 µg mL^−1^; DOX.HCl: 1.0 µg mL^−1^; NAC: 1 mm. b) Relative tumor volume and c) tumor growth inhibition (TGIs) treated with different groups (*n* = 5, ^**^ *p* < 0.01, ^*^ *p* < 0.05). d) Histological and immunohistochemical images of tumor tissue were detected by H&E, Ki‐67, and TUNEL assays (brown: Ki‐67‐positive cells, blue: TUNEL‐positive cells. Scale bar:100 µm).

It is worth noting that the effect of activated autophagy on cancer cells could be either pro‐survival or pro‐death. The cytotoxicity of different treatments was then investigated after adding an autophagy inhibitor, 3‐methyladenine (3‐MA). Adding 3‐MA alone does not induce additional cytotoxicity. However, the cytotoxicity of CeO_2_/Cu‐W and CeO_2_/Cu‐O nanoparticles decreases after autophagy is inhibited by 3‐MA (Figure S15, Supporting Information). Then, the assessment of whether CeO_2_/Cu nanoparticles at a noncytotoxic concentration (100 µg mL^−1^) would impact the cancer cell death induced by doxorubicin (DOX) was performed.^[^ ^31^ ^]^ As shown in **Figure** **8**a, the combination of CeO_2_/Cu‐W and DOX enhances more than 20% of cell death compared to the treatment with DOX, probably due to the autophagic death elicited by CeO_2_/Cu‐W. Furthermore, when NAC is added before the addition of CeO_2_/Cu‐W nanoparticles, the cell death rate is pronouncedly decreased, supporting the chemo‐sensitization effect caused by autophagy. However, when NAC is added after the addition of CeO_2_/Cu‐W nanoparticles, a negligible reduction in the chemo‐sensitization effect is observed since autophagy is induced by the nanoparticles.

Finally, the in vivo synergistic antitumor effect of CeO_2_/Cu nanoparticles and DOX was assessed in the Balb/c nude mice bearing MDA‐MB‐231 tumors through i.v. injection of DOX with a dose of 5 mg kg^−1^ and intratumoral injection of CeO_2_/Cu nanoparticles (5 mg kg^−1^) every 4 days for four times. As presented in Figure 8b, the treatment with CeO_2_/Cu‐W nanoparticles slightly inhibits tumor growth. The tumor volumes in the mice increase by 6 times after DOX treatment. Encouragingly, the treatment with CeO_2_/Cu‐W nanoparticles in combination with DOX has a synergistic antitumor effect, resulting in 3 times smaller of the tumor volume and the tumor inhibit rate (TGI) reaches up to 81.6% (Figure 8c). Furthermore, immunohistochemical studies reveal the fewest Ki67‐positive proliferative cells as well as the most TUNEL‐positive apoptotic cells in the tumor tissue with the treatment of CeO_2_/Cu‐W nanoparticles plus DOX (Figure 8d). Hematoxylin and eosin (H&E) results show remarkable damage to the tumor tissues after the treatment with CeO_2_/Cu‐W nanoparticles plus DOX, compared with compact and superabundant tumor cells in the saline‐treated group. Our results mutually confirm that CeO_2_/Cu nanoparticles combined with DOX could have a potent synergistic effect on the eradication of cancer cells. Consistent with previous data in vitro, the synergistic antitumor effect in vivo is not simply due to an additive effect of two treatments, which provides a promising strategy for combination therapy of multiple types of cancer by promoting the autophagy level. Moreover, the body weight of the mice treated with CeO_2_/Cu nanoparticles gathered during the treatment period displays a slight increase (Figure S16, Supporting Information), sharing a similar trend with that of the saline‐treated group, which indicates that intratumoral injection of CeO_2_/Cu nanoparticles does not induce distinct systemic toxicity.

## 3 Conclusion

In this study, CeO_2_/Cu are reported as a biomimetic SOD to transform O_2_^∙‐^ → H_2_O_2_ and peroxidase (POD) to realize H_2_O_2_ → ∙OH. Importantly, the artificial enzyme of CeO_2_/Cu with a wire shape promotes direct alteration of endogenous ROS to the target molecule (∙OH). Particularly, it can be applied to the intervention of intracellular ROS in cancer cells. With superior SOD‐like and POD‐like catalytic efficiencies, the wire‐shaped CeO_2_/Cu nanoparticles exhibit the best antitumor effect and improve the antitumor drug efficiency. The work offers a promising strategy for manipulating oxygen vacancy formation and the structural shape of catalytic inorganic nanoparticles to regulate the level of intracellular ROS.

## Conflict of Interest

The authors declare no conflict of interest.

## Supporting information

Supporting Information

## Data Availability

Research data are not shared.
