# Supplementary material for: Unraveling Ros Conversion Through Enhanced Enzyme‐Like Activity with Copper‐Doped Cerium Oxide for Tumor Nanocatalytic Therapy
Source: Adv Sci (Weinh). 2023 Dec 31;11(11):2307154. doi: 10.1002/advs.202307154 (PMC10953536; doi:10.1002/advs.202307154)
Supplement: Supplementary file 1 — Supporting Information [file ADVS-11-2307154-s001.pdf]

ADVANCED SCIENCE
Open Access

Supporting Information

for *Adv. Sci.*, DOI 10.1002/advs.202307154

Unraveling Ros Conversion Through Enhanced Enzyme-Like Activity with Copper-Doped Cerium Oxide for Tumor Nanocatalytic Therapy

*Zhengxiang Gu, Dan Zhong, Xingyu Hou, Xuelian Wei, Caikun Liu, Yechuan Zhang, Zhenyu Duan, Zhongwei Gu, Qiyong Gong and Kui Luo**

Supporting Information

Unraveling ROS conversion through enhanced enzyme-like activity with copper-doped cerium oxide for tumor nanocatalytic therapy

Zhengxiang Gu,[a†] Dan Zhong,[a†] Xingyu Hou,[a] Xuelian Wei,[a] Caikun Liu,[c] Yechuan Zhang,[d] Zhenyu Duan,[a] Zhongwei Gu,[a] Qiyong Gong,[a,b] Kui Luo[a,b,*]

[a] Department of Radiology, Huaxi MR Research Center (HMRRC), Frontiers Science Center for Disease-Related Molecular Network, State Key Laboratory of Biotherapy, West China Hospital, Sichuan University, Chengdu 610041, China.

[b] Functional and molecular imaging Key Laboratory of Sichuan Province, and Research Unit of Psychoradiology, Chinese Academy of Medical Sciences, Chengdu, 610041, China.

[c] National Engineering Research Center for Biomaterials, Sichuan University, 29 Wangjiang Road, Chengdu 610064, China

[d] School of Chemistry and Materials Science, Nanjing Normal University, Nanjing, 210023, China.

[†] Z. G. and D. Z contributed equally to this work.
*Corresponding authors.
Prof. Luo is to be contacted at Tel./fax: +86 28 85423622.
E–mail address: luokui@scu.edu.cn

Content

1. Experimental Section **3**

1.1 Structure characterizations **3**

1. 2 Material synthesis **3**

1.2.1 *Synthesis of CeO_2 wires (CeO_2-W) and cubes (CeO_2-C).* 3

1.2.2 *Synthesis of CeO_2 octahedrons (CeO_2-O).* 3

1.2.3 *Preparation of CeO_2/Cu catalysts.* 4

1.3 Calculation details **4**

1.4 Details of POD and SOD-like measurement (Figure 4) **4**

1.5 Acridine orange (AO) staining **5**

1.8 Cellular uptake **5**

1.9 Cytotoxicity Testing **6**

1.10 Reactive oxygen species (ROS) measurement **6**

1.11 In vivo antitumor efficacy **6**

1.12 Statistical analysis **6**

2. Supplementary Results **7**

2.2 Supplementary Figures **7**

References **14**

1. Experimental Section

1.1 Structure characterizations

All materials were of analytical grade and used without further purification.

A field emission scanning electron microscopy (FESEM; ZEISS-Merlin), a transmission electron microscopy (TEM, JEOL-2010) with energy dispersive X-ray spectroscopy (EDX), and a high-resolution TEM (HRTEM, JEOL-2010) were used to characterize the morphology and composition of samples. XRD curves of samples were recorded on Rigaku at 40 kV and 40 mA, and X-ray photoelectron spectroscopy (XPS) curves were obtained on a PHI Quantera SXM (ULVAC-PHI) instrument to determine the compositions and the valence states of the elements in the samples. All electrochemical measurements were performed on a CHI 760E electrochemical work station with a typical three-electrode setting at room temperature. A graphite rod and an Ag/AgCl electrode were selected as a counter and reference electrode, respectively. The self-supporting array grown on carbon cloth (1×1 cm, mass loading ~ 2.5 mg/cm^{-2}) was directly used as a working electrode. The electrochemical data were collected in an electrolyte of 1.0 M KOH.

1. 2 Material synthesis

1.2.1 *Synthesis of CeO_2 wires (CeO_2-W) and cubes (CeO_2-C).*

Generally, a solution composed of 3.472 g $Ce(NO_3)_3 \cdot 6H_2O$ (Aladdin) and 20 mL deionized (DI) water was mixed with the other solution composed of 38.4 g NaOH and 140 mL DI water. After the obtained solution was continually stirred for 0.5 h, it was moved to a 250 mL stainless steel autoclave with a Teflon liner, followed by hydrothermal treatment at 100 ℃ for 24 h to obtain CeO_2-W, and at 180 ℃ for 24 h to obtain CeO_2-C. The precipitation was then purified by centrifugation. After it was rinsed using DI water (until pH = 7) and pure ethanol three times, the final ceria samples were obtained by drying at 80 ℃ for 8 h and calcination in air at 400 ℃ for 4 h.

1.2.2 *Synthesis of CeO_2 octahedrons (CeO_2-O).*

A solution composed of 1.716 g $Ce(NO_3)_3 \cdot 6H_2O$ and 20 mL DI water was mixed with the other solution composed of 0.015 g Na_3PO_4 and 140 mL DI water. After the obtained solution was continually stirred for half an hour, it was moved into a 250 mL autoclave and hydrothermally treated at 170 ℃ for 10 h. Finally, the formed solids were separated, dried and calcined by following the same procedure as CeO_2-W.

***1.2.3** Preparation of CeO_2/Cu catalysts.*

The CeO_2-supported Cu catalysts were prepared by an incipient wetness impregnation method. The loadings of the Cu metal for these catalysts were kept to be 4.0 wt%. After impregnation, the samples were dried slowly in vacuum at 40 ℃ and then at 120 ℃ in an oven for 12 h. These dried samples were calcined in N_2 at 300 ℃ for 5 h and finally reduced in H_2 at 120 ℃ for 1 h and then at 250 ℃ for 0.5 h at a temperature ramping rate of 5 ℃ min^{-1}. The Cu contents in these sample was analyzed by ICP (TJA IRIS 1000), which were 4.03 wt% for CeO_2/Cu-W, 4.08 wt% for CeO_2/Cu-C, and 4.15 wt% for CeO_2/Cu-O, respectively.

1.3 Calculation details

In this work, the DFT calculations were performed using the Vienna *ab initio* simulation package (VASP).[1, 2] The projection augmented wave (PAW) method with a frozen-core approximation was used for the description of the interaction between electrons and ions,[3, 4] and the electron exchange and correlation were treated within the generalized gradient approximation with Perdew-Burke-Ernzerhof (GGA-PBE).[5, 6] The cut-off energy is set at 450 eV. The Brillouin zone integrations were approximated by k-points chosen using the Monkhorst-Pack grid. A self-consistent field energy tolerance was set at 10^{-4} eV, and the maximum force tolerance of 0.02 eV/Å was used for geometries optimization. The DFT+U approach was used, and the value of U=5 eV was used for Ce 4f electrons.[7]

The formation energy of an O vacancy can be calculated by

$$Ef_{Vo}=E(CeO_{2\text{-}Vo})+1/2E(O_2)\text{-}E(CeO_2))$$

where $E(CeO_{2\text{-}Vo})$, $E(CeO_2)$ and $E(O_2)$ are the energy for the CeO_2(111) with an O vacancy, pure CeO_2(111) and O_2 molecule in the gas phase.

1.4 Details of POD and SOD-like measurement (Figure 4)

Dual mimetic enzyme activity. (a-c) Temporal absorbance variations at a wavelength of 650 nm in different shaped CeO_2/Cu/TMB/H_2O_2 systems at pH of 4.4, 5.0 and 6.0. The samples were prepared from a mixture of 0.15 M H_2O_2, 0.2 mg TMB, and 1 ml buffer (pH 4.4, pH 5.0 and pH 6.0) at a concentration of CeO_2/Cu -W, CeO_2/Cu-C or CeO_2/Cu-O of 10 μg. (d-f) Normalized temporal absorbance variations at 540 nm wavelength in different shaped CeO_2/Cu/NBT/Riboflavin-photogenerated $O_2^{\bullet-}$ systems at pH of 6.0, 7.4 and 8.4. The samples were prepared from a mixture of 23 μg Riboflavin, 20 μmol EDTA, 1.88 mg NBT and 3 ml PBS buffer (pH 6.0, pH 7.4 and pH 8.4) at a concentration of CeO_2/Cu of 20 μg. (g) ESR signal of DMPO/ $O_2^{\bullet-}$ in two reaction systems of (Xan + XOD)/DMPO and CeO_2/Cu/(Xan + XOD)/DMPO. Xan: 0.4 mM, XOD: 8 U, DMPO: 5 mM, and CeO_2/Cu: 20 μg. (h) ESR signal

of DMPO/•OH in two reaction systems of H_2O_2/DMPO and CeO_2/Cu /H_2O_2/DMPO. H_2O_2: 0.5 mM, DMPO: 50 mM, CeO_2/Cu: 10 μg/mL, buffer: pH 6.0, and reaction time: 5 min.

1.5 Acridine orange (AO) staining

MDA-MB-231 cells were seeded in 8-well chambered coverglass system and then incubated with CeO_2/Cu nanoparticles (100 μg mL^{-1}). As a positive control, rapamycin (Rapa) was added at a final concentration of 50 nM at the same time. After 24 h, the cells were stained with AO (1 μM) for 10 min at 37 °C and detected by a confocal laser scanning microscope (CLSM, N-SIM S, Nikon, Japan) with an excitation wavelength at 488 nm and emission wavelengths from 505 to 525 nm (green) and from 610 to 640 nm (red). To quantitatively determine the red/green ratio in AO staining cells, these MDA-MB-231 cells after treatment with CeO_2/Cu nanoparticles were harvested for flow cytometry analysis. The mean red/green fluorescence ratio in the cells was calculated using FlowJo software. All the experiments were performed in triplicate.

1.6 Observation of fluorescent LC3 dot formation

MDA-MB-231 cells were seeded in confocal dishes at a density of 1×10^4 per well. After reaching 30% confluence, the cells were transfected with mCherry-GFP-LC3 adenovirus at a multiplicity of infection (MOI) value of 50. After infection for 12-16 h, the virus-containing media were removed and replaced with 400 μL of fresh media.

To analyze the autophagic flux, mCherry-GFP-LC3-expressing MDA-MB-231 cells were treated with different CeO_2/Cu nanoparticles for 24 h and imaged with a CLSM. The fluorescent LC3 dot formation was quantified by counting 500 cells and expressed as the ratio of GFP positive cells and mCherry positive cells over the total number of cells.

1.7 Bio-transmission electron microscopy (Bio-TEM)

MDA-MB-231 cells were seeded in 6-well plates. After reaching 50% confluence, the cells were treated with CeO_2/Cu nanoparticles for 24 h. Cells were collected by trypsinization, washed with PBS three times and then fixed in 0.01 M PBS (pH 7.4) containing 0.5% glutaraldehyde for 10 min at 4 °C. After centrifugation at 12000 g for 15 min, the cells pellets were fixed in 3% glutaraldehyde solution at 4 °C and sent to Chengdu Lilai Biotechnology Co. Ltd. (Chengdu, China) for dehydration, embedding, sectioning and further imaging with a H-600IV TEM (Hitachi group, Japan).

1.8 Cellular uptake

MDA-MB-231 cells (2×10^5 per well) were seeded in 6-well plates and cultured for 24 h. The cells were treated with CeO_2/Cu nanoparticles (50 μg mL^{-1}) for 6 h. After washing with PBS 3 times, the cells were collected, digested with 70% w/v nitric acid and heated to 120 °C

to remove the vapors. After cooling to room temperature, the residues were dissolved in 5 mL of deionized water and the samples were filtered through a 0.45 μm hydrophilic membrane. ICP-MS was performed to measure the content of cerium.

1.9 Cytotoxicity Testing

The cytotoxicity of CeO_2/Cu nanoparticles was assessed by the CCK-8 assay. MDA-MB-231 cells were seeded in 96-well plates (5×10^3 cells per well) and cultured for 24 h. After incubation with CeO_2/Cu nanoparticles for 24 or 48 h, the cells were washed with PBS and incubated with 100 μL of FBS-free medium containing 10% CCK-8 reagent (Dojindo Laboratories, Japan) for another 2 h. The absorbance at 450 nm was measured using a microplate reader. The relative cell viability was calculated according to the equation: cell viability = $(OD_{sample}-OD_{background})/(OD_{control}-OD_{background}) \times 100\%$.

1.10 Reactive oxygen species (ROS) measurement

To probe the generation of ROS inside cells, MDA-MB-231 cells were seeded in 96-well plates and treated with PBS, CeO_2/Cu-W, CeO_2/Cu-C or CeO_2/Cu-O nanoparticles for 3, 6, 12 and 24 h after they reached a confluence of about 70%. The cells were then exposed to FBS-free medium containing 10 μM DCFH-DA for 30 min and the absorbance at 510 nm was read via a microplate reader. The cells treated with PBS was used as a control. To directly monitor the intracellular ROS generation process, the cells were seeded on glass-bottomed dishes and treated with CeO_2/Cu nanoparticles for 12 h. After staining with DCFH-DA for 30 min and Hoechst 33342 for 15 min, the cells were imaged with a CLSM at an excitation of 488 nm and an emission of 505-525 nm.

1.11 In vivo antitumor efficacy

A tumor model was established by subcutaneously injecting 1×10^6 MDA-MB-231 cells into the right flank of BALB/c nude mice (4 weeks, female). When the tumor volume reached about 50 mm^3, the mice were randomly divided into 8 groups. DOX·HCl were intravenously administered at a dose of 5 mg DOX per kg body weight and CeO_2/Cu nanoparticles were intratumorally injected at a dose of 10 mg per kg body weight on day 0, 4, 8 and 12. The tumor volume ($V = lw^2/2$, w: width, l: length) and body weight were recorded every 2 days by a caliper. The significant difference was evaluated by the student T test. At the end of the treatment course, the mice were sacrificed and the tumors were excised for immunohistochemistry analysis and hematoxylin and eosin (H&E) staining. All animal procedures were performed in accordance with the Guidelines for Care and Use of Laboratory Animals of Sichuan University and the experiments were approved by the ethics committee of Sichuan University.

1.12 Statistical analysis

Data are shown as mean ± standard deviation (SD). Statistical analysis was carried out using GraphPad Prism Software. For two groups, statistical significance was analyzed using two-tailed Student's t-tests. In comparisons among multiple groups, a one-way ANOVA with Tukey's post-hoc test was used for between-group comparisons. In all cases, significant difference was defined as $p \leq 0.05$.

2. Supplementary Results

2.2 Supplementary Figures

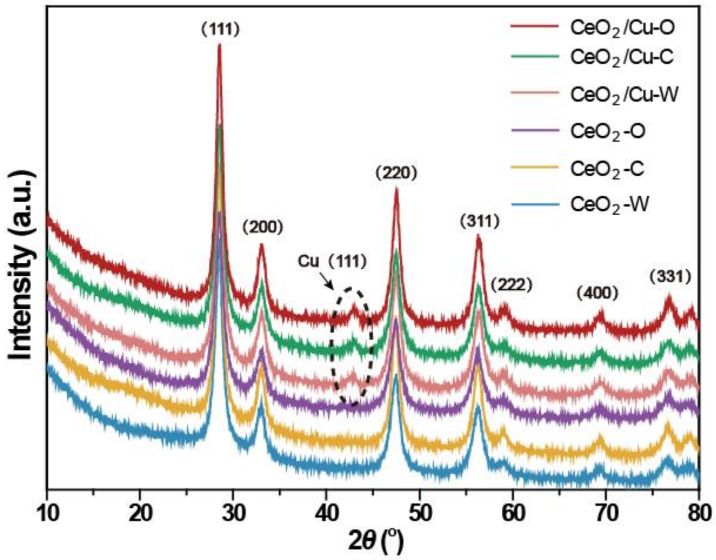

Figure S1. XRD patterns of as synthesized CeO_2 and CeO_2/Cu.

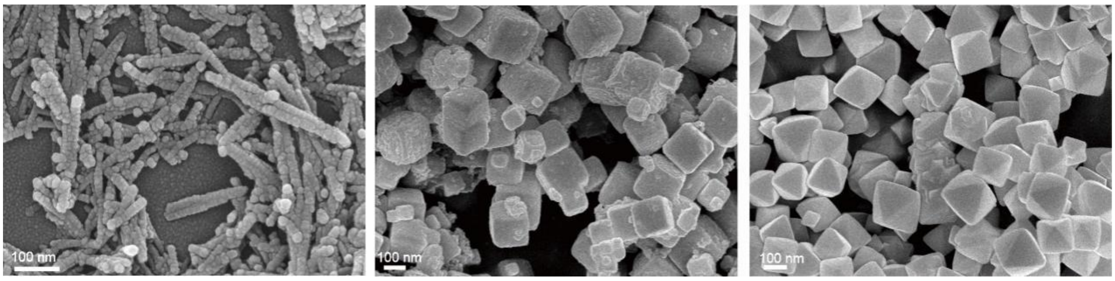

Figure S2. SEM images of CeO_2 with different morphology.

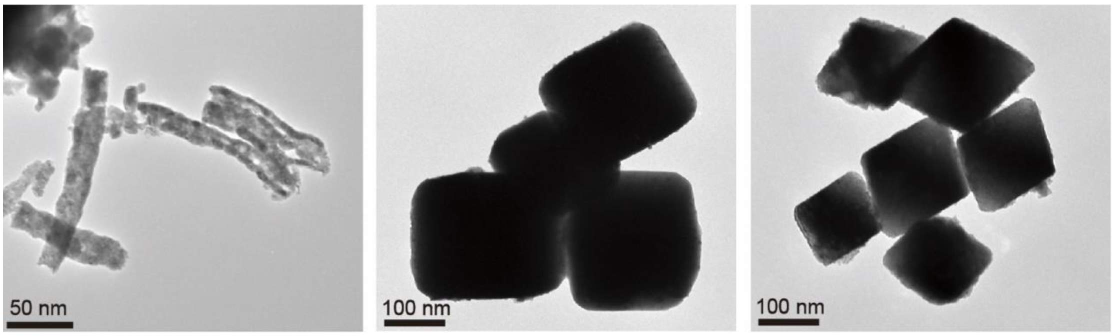

Figure S3. TEM images of CeO_2 with different morphology.

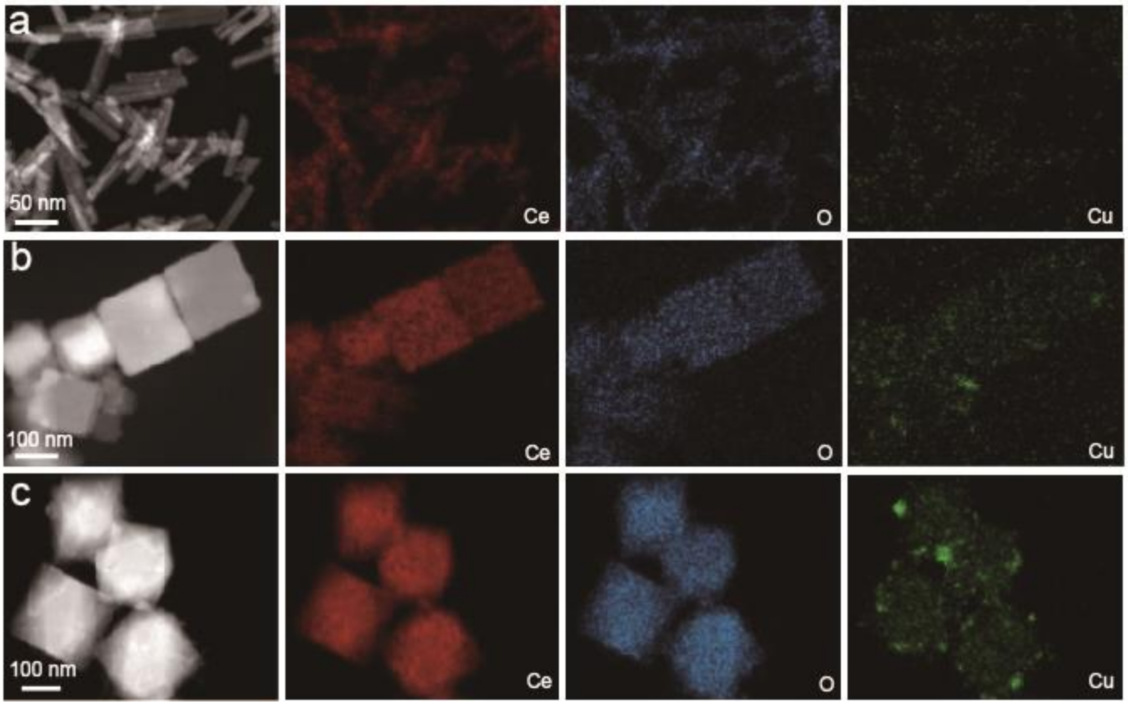

Figure S4. HAADF-STEM images and corresponding EDX elemental mappings of catalysts: (a) CeO_2/Cu-W, (b) CeO_2/Cu-C, and (d) CeO_2/Cu-O.

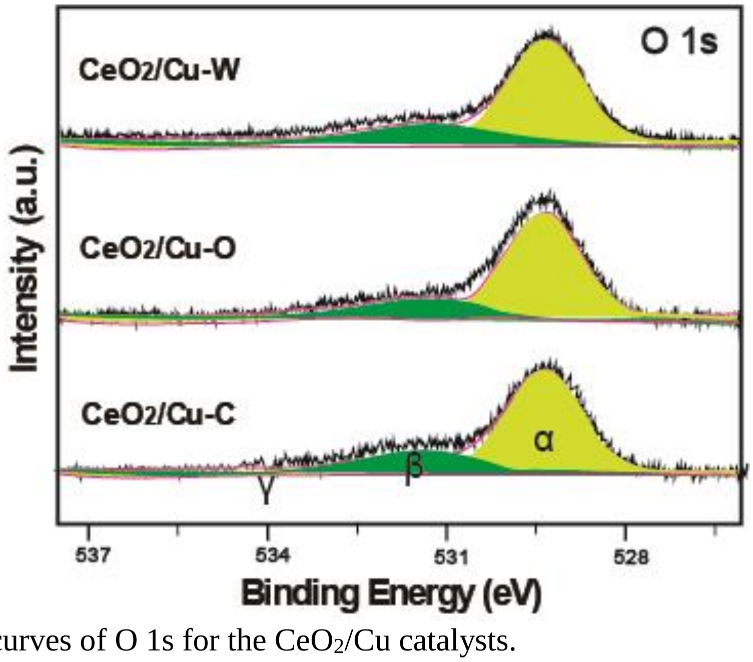

Figure S5. XPS curves of O 1s for the CeO_2/Cu catalysts.

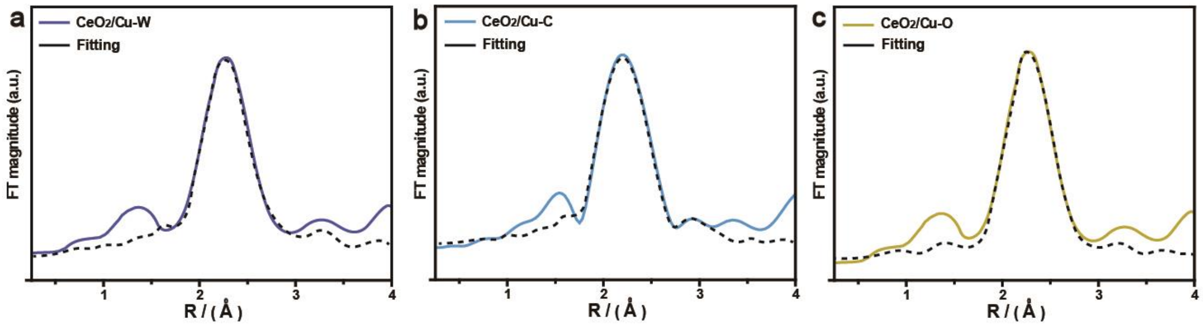

Figure S6. (a−c) Cu K-edge FT-EXAFS spectra in the R-space of the CeO_2/Cu samples.

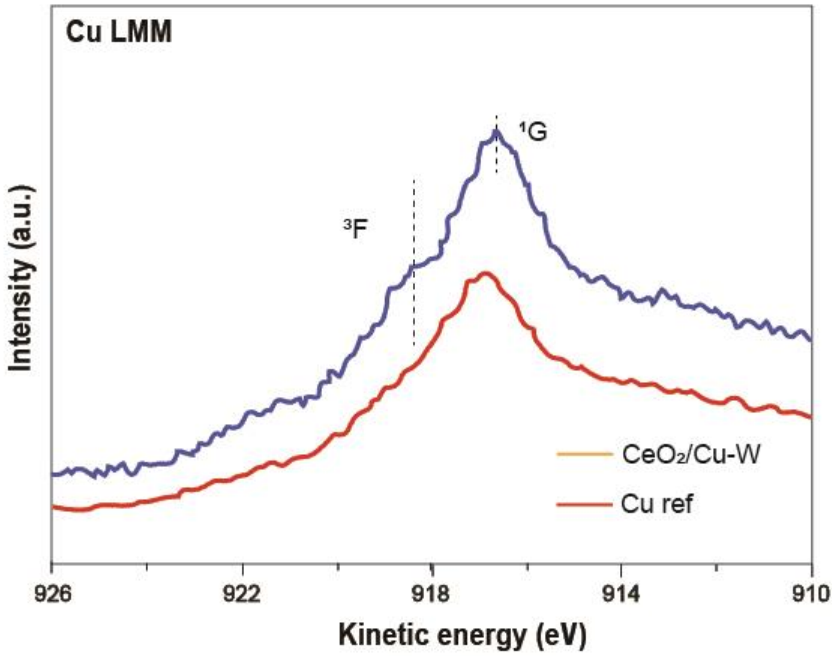

Figure S7. Cu LMM Auger spectra of the CeO_2/Cu-W sample.

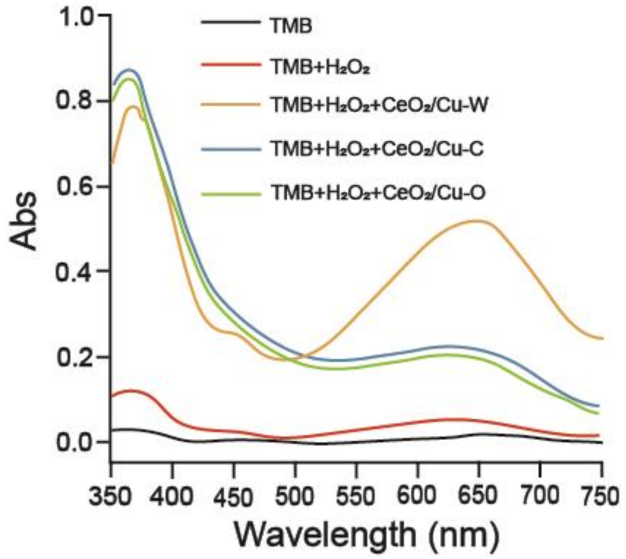

Figure S8. The absorption spectra in the presence of 5 μg CeO_2/Cu, 1% H_2O_2 and 1.0 mmol/mL TMB in the pH 6.0 PBS buffer solution.

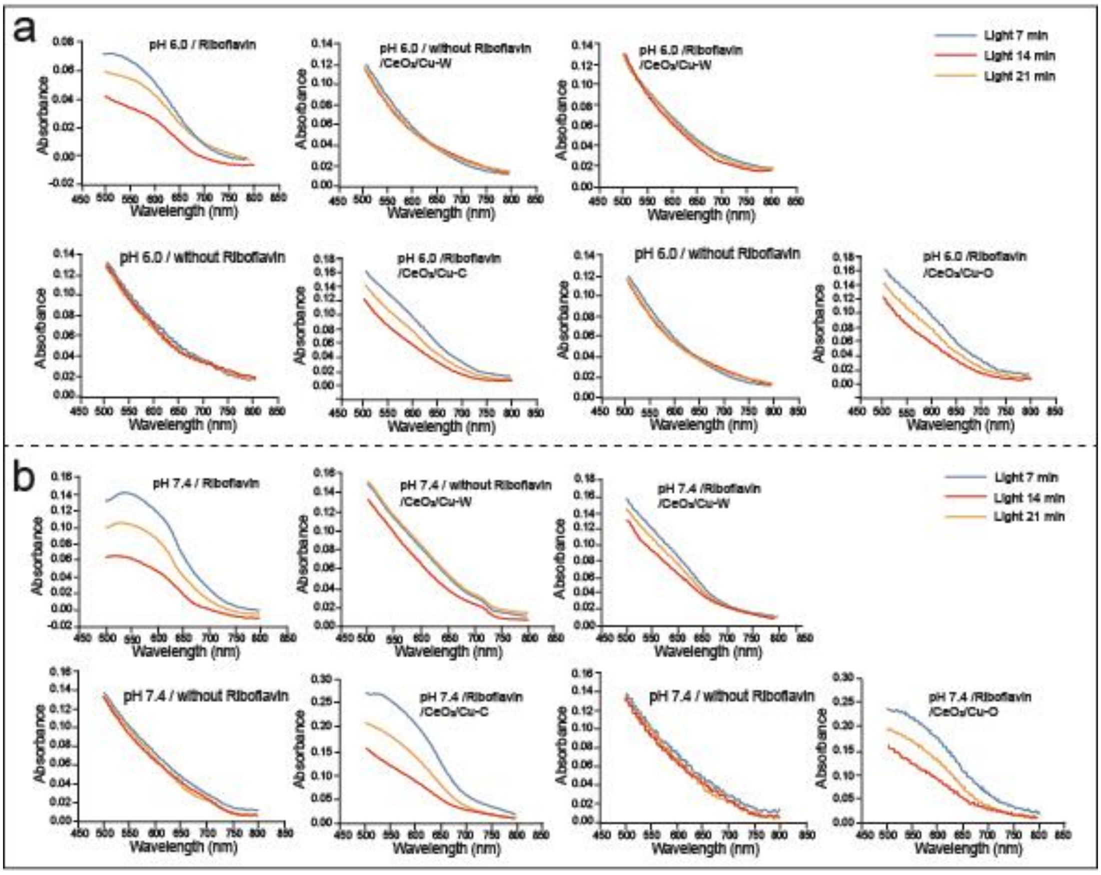

Figure S9. (a, b) The feasibility of applying the above detection method at the conditions of pH 6.0 and pH 7.4.

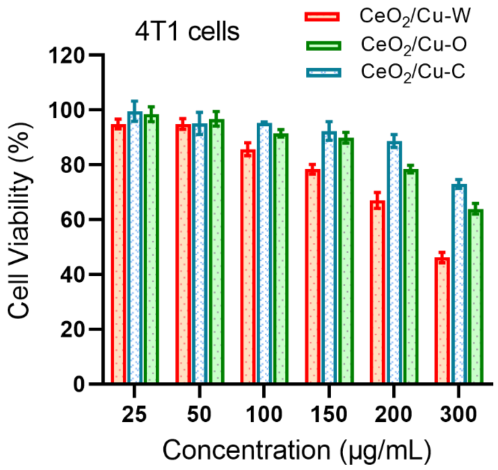

Figure S10. Cell viabilities of 4T1 cells after treatment with CeO_2/Cu nanoparticles for 24 h. Data are presented as mean ± SD ($n = 5$).

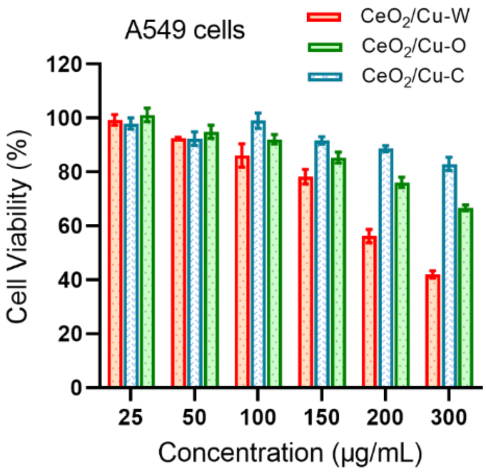

Figure S11. Cell viabilities of A549 cells after treatment with CeO_2/Cu nanoparticles for 24 h. Data are presented as mean ± SD (*n* = 5).

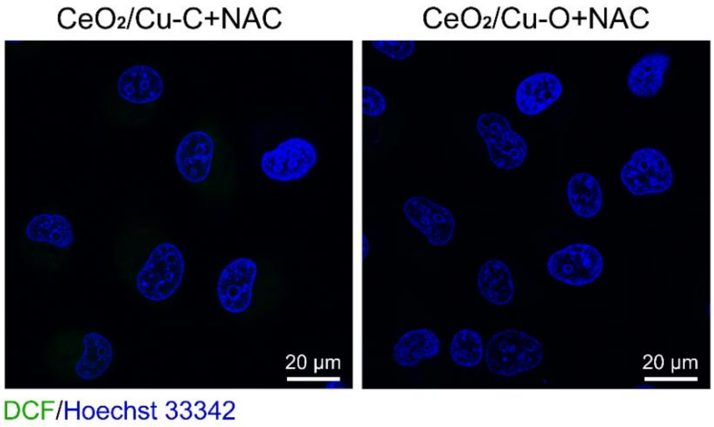

Figure S12. Confocal images of intracellular ROS in cells after incubation for 12 h with CeO_2/Cu nanoparticles in the presence *N*-acety-L-cysteine (NAC), a ROS scavenger, NAC was added two hours before the addition of Nano-C60. DCF fluorescence (green) for intracellular ROS and Hoechst 33342 (blue) for cell nuclei.

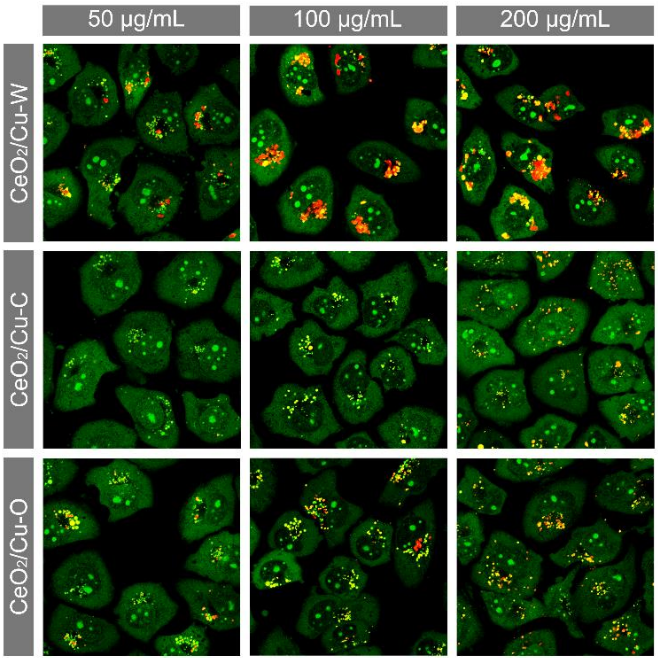

Figure S13. CLSM images of AO-stained MDA-MB-231 cells after incubation with different concentration of CeO_2/Cu-W, CeO_2/Cu-C or CeO_2/Cu-O nanoparticles for 24 h.

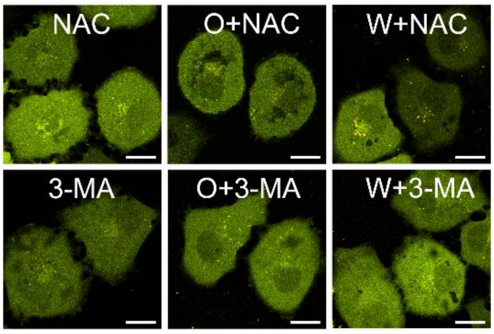

Figure S14. CLSM images of mCherry-GFP-LC3-expression cells after incubation with different formulations for 24 h.

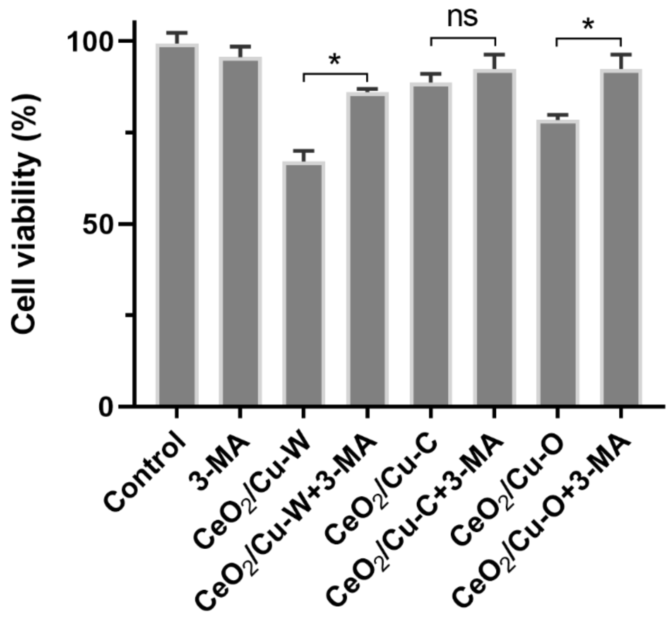

Figure S15. Cell viabilities of MDA-MB-231 cells after treatment with various combinations for 24 h. CeO_2/Cu nanoparticles: 200 μgmL^{-1}; 3-MA: 0.5 mM. Data are presented as mean ± SD (n = 5, ns nonsignificant, *$p < 0.01$).

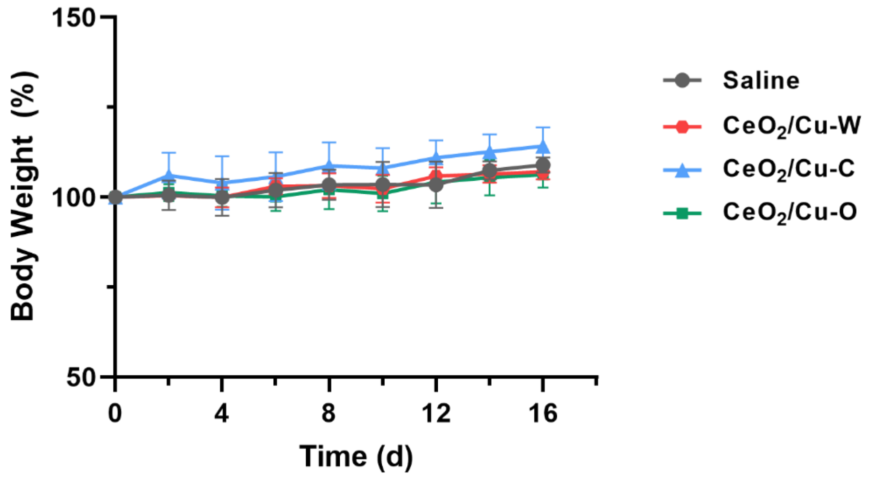

Figure S16. Body weight changes of 4T1 tumor-bearing mice after different treatments.

Supplementary tables

Table S1. Cu contents in different samples determined by ICP-AES.

Sample ID	Reported Conc (Samp)	Samp Units	%
CeO_2/Cu-W	0.842	mg/L	4.03
CeO_2/Cu-C	0.846	mg/L	4.08
CeO_2/Cu-O	0.851	mg/L	4.15
Cu standard	20.77	mg/L	100

Table S2. Binding energies of deconvoluted plots of Ce 3d spectra for the catalysts along with the contents of Ce^{3+} over the catalyst surfaces.

Sample ID	Ce $3d_{5/2}$ (eV)					Ce $3d_{3/2}$ (eV)				
	ν_o	ν	ν'	ν''	ν'''	μ_o	μ	μ'	μ''	μ'''
CeO_2 /Cu-W	880.5	882.7	885.3	888.9	898.5	899.2	900.8	903.5	907.3	916.7
CeO_2 /Cu-C	880.5	882.7	885.2	888.9	898.5	899.2	900.8	903.4	907.3	916.7
CeO_2 /Cu-O	880.5	882.7	885.2	888.9	898.5	899.2	900.8	903.4	907.3	916.7

References

[1] G. Kresse, J. Furthmuller, *Comp. Mater. Sci.* **1996**, *6*, 15.
[2] G. Kresse, J. Furthmuller, *Phys. Rev. B* **1996**, *54*, 11169.
[3] P.E. Blochl, C.J. Forst, J. Schimpl, *B. Mater. Sci.* **2003**, *26*, 33.
[4] G. Kresse, D. Joubert, *Phys. Rev. B* **1999**, *59*, 1758.
[5] Perdew, Yue, *Phys. Rev. B* **1986**, *33*, 8800.
[6] J.P. Perdew, K. Burke, M. Ernzerhof, *Phys. Rev. Lett.* **1997**, *78,* 1396.
[7] H. Y. Li, H. F. Wang, X. Q. Gong, Y. L. Guo, Y. Guo, G. Lu, P. Hu, *Phys. Rev. B* **2009**, *79*, 193401.
